# “You know, we’re all human beings”: A qualitative study on the perceived needs of people experiencing chronic pain with regard to physiotherapy services

**DOI:** 10.1080/24740527.2026.2615474

**Published:** 2026-03-04

**Authors:** Jonathan Gervais-Hupé, Arthur Filleul, Kadija Perreault, Isabelle Gaboury, Timothy H. Wideman, Céline Charbonneau, Fatiha Loukili, Anne Hudon

**Affiliations:** a School of Rehabilitation, Faculty of Medicine, Université de Montréal, Montreal, Quebec, Canada; b Centre for Interdisciplinary Research in Rehabilitation of Greater Montreal (CRIR) of the Institut universitaire sur la réadaptation en déficience physique de Montréal (IURDPM), Centre intégré universitaire de santé et de services sociaux du Centre-Sud-de-l’Ile-de-Montréal (CCSMTL), Montreal, Quebec, Canada; c Centre de recherche en éthique (CRÉ), Montreal, Quebec, Canada; d UFR de Médecine et de Pharmacie, Université Grenoble Alpes, Grenoble, France; e Center for Interdisciplinary Research in Rehabilitation and Social Integration (Cirris), Centre Intégré Universitaire de Santé et de services sociaux de la Capitale-National, Quebec City, Quebec, Canada; f École des sciences de la réadaptation, Faculté de médecine, Université Laval, Quebec City, Quebec, Canada; g Department of Family Medicine and Emergency Medicine, Faculty of Medicine and Health Sciences, University of Sherbrooke, Longueuil, Quebec, Canada; h School of Physical and Occupational Therapy, Faculty of Medicine and Health Sciences, McGill University, Montreal, Quebec, Canada; i Centre for Interdisciplinary Research in Rehabilitation of Greater Montreal (CRIR), Centre intégré universitaire de santé et de services sociaux du Centre-Ouest-de-l’Ile-de-Montréal, Montreal, Quebec, Canada; j Association québécoise de la douleur chronique, Montreal, Quebec, Canada; k Association des personnes vivant avec de la douleur chronique, Gatineau, Quebec, Canada

**Keywords:** Perceived needs, chronic pain, physiotherapy, therapeutic relationship, health care environment, health care organization, patientcentered care

## Abstract

**Background:**

Chronic pain has many deleterious effects on the lives of those living with it. Though understanding the needs of people living with chronic pain is crucial to providing patient-centered care, current knowledge remains limited regarding the needs of this population in relation to the physiotherapy services they regularly use.

**Aims:**

This study aimed to understand the perceived needs of people living with chronic pain regarding physiotherapy services in Quebec, Canada.

**Methods:**

This qualitative study followed an interpretative description methodology. Semistructured individual interviews were conducted with adults living with chronic pain who had used physiotherapy services in any type of setting. Transcripts were analyzed using reflexive thematic analysis following an inductive approach with the constant comparative method.

**Results:**

Among the 27 participants, the majority were White women living in urban areas who were highly educated. They mainly used physiotherapy services in private clinics for musculoskeletal chronic pain. Four overarching themes related to patients’ perceived physiotherapyrelated needs were identified: (1) being respected in an empathetic human-to-human relationship; (2) obtaining the care you need; (3) desiring a warm, welcoming, and tailored environment; and (4) feeling the organization practices and policies are adapted to persons living with chronic pain.

**Conclusion:**

Our findings show that the needs of people living with chronic pain are multiple, deeply interconnected, and shaped by organizational and systemic contexts. Improving the responsiveness of physiotherapy services therefore requires moving beyond individual-level strategies to address these broader forces. A comprehensive, systemwide approach is essential to meaningfully meet patients’ needs.

## RÉSUMÉ

**Contexte :** La douleur chronique entraîne de nombreux effets délétères sur la vie des personnes qui vivent avec celle-ci. Bien que la compréhension des besoins des personnes vivant avec la douleur chronique soit essentielle à la prestation de soins centrés sur le patient, les connaissances actuelles demeurent limitées quant à leurs besoins en lien avec les services de physiothérapie qu’elles utilisent régulièrement.

**Objectif :** Cette étude visait à comprendre les besoins perçus des personnes vivant avec la douleur chronique à l’égard des services de physiothérapie au Québec, Canada.

**Méthodes :** Cette étude qualitative s’est appuyée sur une méthodologie de description interprétative. Des entretiens individuels semi-structurés ont été menés auprès d’adultes vivant avec la douleur chronique ayant utilisé des services de physiothérapie, quel que soit le contexte de prestation. Les transcriptions ont été analysées au moyen d’une analyse thématique réflexive selon une approche inductive, fondée sur la méthode comparative constante.

**Résultats :** Parmi les 27 participants, la majorité étaient des femmes blanches vivant en milieu urbain et hautement instruites. Elles utilisaient principalement des services de physiothérapie en clinique privée pour des douleurs chroniques musculosquelettiques. Quatre thèmes généraux relatifs aux besoins perçus par les patients à l’égard de la physiothérapie ont été dégagés : (1) être respecté dans une relation empathique d’humain à humain ; (2) recevoir les soins dont on a besoin; (3) désirer un environnement chaleureux, accueillant et personnalisé; et (4) percevoir que les pratiques et les politiques organisationnelles répondent aux besoins des personnes vivant avec la douleur chronique.

**Conclusion :** Nos résultats montrent que les besoins des personnes vivant avec la douleur chronique sont multiples, profondément interreliés et façonnés par des contextes organisationnels et systémiques. L’amélioration de la capacité de réponse des services de physiothérapie nécessite donc d’aller au-delà des stratégies centrées uniquement sur l’individu afin de tenir compte de ces dynamiques plus larges. Une approche globale à l’échelle du système est essentielle pour répondre de manière significative aux besoins des patients.

## Introduction

Chronic pain affects one in five people worldwide.^1,^^2^ It negatively impacts people’s physical, psychological, and social well-being and entails staggering societal costs.^3–5^ In addition to the intense, disabling, and unpredictable nature of pain,^6^ people living with chronic pain are more prone to depression, anxiety, and substance use disorders.^7,8^ Chronic pain also affects people’s social life; for instance, contributing to higher perceived rejection rates, lower quality relationships, and social isolation.^9^ Furthermore, there is ample evidence of stigma experienced by people with chronic pain, who often feel disbelieved and misunderstood by family, friends, and colleagues, as well as by health care professionals.^10^

Among the many nonpharmacological interventions recommended to help people living with chronic pain, physiotherapy is one of the most frequently used.^1,11^ As with other health services, there have been calls for physiotherapy services to be patient centered; that is, to be respectful of, and responsive to, patients’ needs, values, and preferences.^12–14^ Though patients’ needs are central to patient-centered care,^15^ the term “need” is complex and remains poorly defined.^16^ Conceptualizing health care needs is particularly challenging, and various theories, models, and categorizations have been proposed to conceptualize health care needs.^17–19^ For example, some authors have described health care needs based on individuals’ ability to benefit from a given health service^19–22^ or according to whether they are normatively defined^17^ or assessed by experts^23,24^ or perceived by individuals to whom health care services are addressed.^23–25^ In this study, we examined the needs perceived and articulated by individuals requiring care, irrespective of whether these needs were currently fulfilled or remained unmet. Expert-assessed needs and patient-perceived needs often differ,^26,27^ and the former are also generally more studied than the latter.^28^ Though no formal definition exists, “perceived needs” relates to the needs felt by individuals to whom health care services are addressed, which include their demands, preferences, or expectations for health care services.^29–31^

Although asking patients about their needs may seem intuitive within a patient-centered approach, clinicians seem more likely to provide care they think could help patients, rather than asking patients about their needs and trying to answer them.^32^ This lack of consideration given to the needs perceived and expressed by people could be the result of a lack of understanding of what needs are^16^ or a misinterpretation of what they mean for the individuals who perceive them.^33^ This is concerning because it can be difficult for some people, particularly the more vulnerable, to have the opportunity to have their needs understood and met.^33^ Therefore, giving people the opportunity to express their needs—for instance, through qualitative studies—is likely to encourage discussion, understanding, and consideration of their needs, which is particularly important for people living with chronic pain. Chronic pain can shape patients’ lived experiences and generate certain needs. For instance, long wait times in the public system may compel some to seek private physiotherapy, which may often be unaffordable, particularly for those already facing financial strain due to disability or job loss.^34,35^ Furthermore, these individuals might also experience frequent episodes of distress and stigmatization related to the health care services provided.^36,37^ These issues reflect the intersection of multiple needs, many of them influenced by the structure and organization of the health care system.

Taking an interest in and listening to patients’ perceived needs is thus crucial for people living with chronic pain. Moreover, fulfilling these needs can positively impact a patient’s satisfaction and quality of life.^30^ Therefore, to offer high-quality, patient-centered services it is essential “to care for patients on their own terms, in their social context, while meeting their needs, (e.g., hours of operation, using telehealth or direct access options, and care that is transferable to a selfmanagement setting) not yours,”^12(p2)^ as mentioned by Cook et al. in reference to physiotherapists.^12^ Taking an active interest in and directly questioning patients about their needs through qualitative research is an effective approach to gaining deeper insights into patients’ perceived needs.^38^ Qualitative research is particularly well suited for this purpose, because it allows for in-depth exploration of individual cases and fosters a richer understanding of each person’s unique lived experience.^39^ This is especially important given the subjective and multifaceted nature of perceived needs, which are often shaped by individuals’ values, beliefs, and personal contexts, particularly in the setting of chronic pain and physiotherapy.

A scoping review was recently conducted by our team to understand the extent of the literature on perceived needs of people living with chronic pain in regard to physiotherapy services.^31^ Based on the results of this review, most studies conducted to date have targeted participants with a specific diagnosis or condition (e.g., low back pain, arthritis, etc.) or a specific physiotherapy intervention (e.g., exercises, selfmanagement, etc.). None of the studies provided an overview of the perceived needs of people living with chronic pain, regardless of diagnosis or intervention, and with regards to physiotherapy services more widely.

Despite recommendations to offer health care services that meet the needs of people living with chronic pain, substantial challenges persist.^40^ For example, in Quebec, the second most populous province in Canada, physiotherapy is regulated by a provincial professional order (Ordre professionnel de la physiothérapie du Québec) that recognizes two professions: physiotherapists and physiotherapy technologists.^41^ These health professionals work across diverse settings in both the public and private sectors. Despite more than 9500 physiotherapy professionals working in the province, people living with chronic pain who seek physiotherapy often encounter multiple barriers. Access to publicly funded, outpatient physiotherapy services is particularly limited, because these services typically require a medical prescription, involve long wait times, and rely on prioritization criteria that tend to disadvantage people living with chronic pain.^42^ Additionally, access to specialized secondary and tertiary services remains restricted, with long delays that can further compromise well-being.^35,43^ As a result, many must turn to the private sector to obtain physiotherapy services, where access can be faster and direct (without a medical prescription) for certain groups of people, yet often costly for others. Moreover, private physiotherapy services seem to mainly target people presenting with acute or subacute musculoskeletal conditions and therefore may not be fully tailored for the needs of people living with chronic pain.^44^ A deeper understanding of the needs of people living with chronic pain, particularly in relation to physiotherapy services, could contribute to enhancing service organization and the quality of care provided.

Thus, this study aimed to understand the perceived needs of people living with chronic pain regarding physiotherapy services in Quebec, Canada.

## Methodology

### Study design

This qualitative study is part of a larger sequential mixed methods project aiming to understand how and to what extent physiotherapy services respond to the needs of people living with chronic pain. Given the complexity of understanding patients’ perceived needs, we chose a qualitative approach to capture the depth and richness of participants’ experiences.^39^ We followed an interpretative description qualitative design.^45–47^ This methodology is particularly well suited for applied health research, because it enables researchers to maintain methodological rigor while acknowledging multiple realities and generating clinically relevant knowledge.^45,47^ This approach enables us to examine phenomena with the goal of identifying themes and patterns among subjective perspectives, while accounting for individual variations.^46^ Aligned with interpretive description, we analyzed participants’ narratives to identify their perceived needs while remaining closely grounded in their words to avoid distorting or overinterpreting these needs. In this regard, perceived needs were considered as a subjective and individual concept that encompasses any person’s demands, preferences, or expectations toward physiotherapy services based on their experiences, beliefs, and values,^31^ regardless of whether they were met or unmet. The study protocol was approved by the Research Ethics Board of the Center for Interdisciplinary Research in Rehabilitation of Greater Montreal (Comité d’éthique de la recherche en réadaptation et en déficience physique du Center de recherche interdisciplinaire en réadaptation du Montréal métropolitain), Montreal, Canada (Project No. 2020–1319).

### Study population and recruitment:

Participants were eligible if they (1) were over 18 years of age; (2) experienced any type of chronic pain (i.e., pain that persists or recurs for more than 3 months^48^); (3) had used physiotherapy services during the previous 3 years in any setting (public or private), provided by physiotherapists or physiotherapy technologists (the two types of professionals recognized by the provincial professional order), in the province of Quebec, Canada; and (4) spoke French or English.

A 3-year period was selected to ensure accurate recall of the experience, while allowing sufficient time for participants to have possibly consulted more than once or to have had adequate exposure to physiotherapy.

Individuals interested in participating in the study who had previously consulted for physiotherapy services in a setting where one of the authors works were excluded.

Purposive sampling using maximum variation was employed to collect diverse perspectives regarding perceived needs.^49^ For example, we tried to include participants from diverse ethnic backgrounds, with varying types of conditions or chronic pain, and who have consulted in physiotherapy in a range of settings. Hence, the recruitment was carried out in two phases. In the first phase, participants were recruited through an invitation sent by email to members of several associations of people living with chronic pain across the province of Quebec and on a Facebook page of physiotherapists and physiotherapy technologists in the province of Quebec. Fifteen interviews were first conducted mainly through convenience sampling, after which a preeligibility questionnaire was used for the second phase of recruitment to ensure maximum variation of the overall sample. A recruitment questionnaire included questions on ethnicity, conditions for which they were treated in physiotherapy, type of care settings they consulted, type of region they lived in, and satisfaction with physiotherapy services.

### Data collection

An interview guide was developed based on the published literature regarding perceived needs related to health care services, more specifically physiotherapy services, as well as on the clinical experience of the first two authors and the two patient partners. The interview questions covered participants’ experiences with pain, physiotherapy, and other health care services, as well as their needs regarding these services. The interview guide consisted of broad questions aimed at exploring participants’ experiences with physiotherapy services, as well as their needs and expectations. For example, it included questions such as, “Could you please describe your experience(s) with physiotherapy services?” and “Could you please describe your needs and expectations in regard to physiotherapy services?” The interview guide also included follow-up questions designed to elicit further detail and clarify participants’ responses. These probes addressed specific aspects of physiotherapy services, including appointment scheduling and management, interactions with the physiotherapist, and the physical environment, among others. The interview guide is available as a Supplementary file. The guide was piloted with three people living with chronic pain who had received physiotherapy services on several occasions. Two of these three people were the patient partners involved in the project (C.C. and F.L.). Following these tests, minor changes were made to the interview guide to make it more comprehensive and improve the flow of the interviews. The interview guide was applied iteratively across interviews, with minor adjustments made as needed.

Twenty-seven interviews were conducted virtually using the Zoom platform (*n* = 21) or by phone (*n* = 6), depending on the participants’ preferences. The audio content of all interviews was recorded on Zoom or using a digital audio recorder. Twenty-one interviews were conducted by the first author (J.G.H.), and the six others were conducted by the second author (A.F.).

#### Sampling

Nineteen women and eight men took part in an interview. Among these, 67% were 45 years old or older, 78% lived in an urban area, 67% had a university degree, and 30% had an income considered to be below the minimum required to meet basic needs, as defined by the provincial government.^50^ In response to the question, “From your perspective, which ethnic or cultural group do you identify with?,” most participants answered White (*n* = 15). Most participants had used physiotherapy services in a private clinic for a chronic musculoskeletal pain condition. Most had received services in more than one setting. Sociodemographic and clinical characteristics of the participants are shown in Table 1.

**Table 1.** Sociodemographic and clinical characteristics of participants (*n* = 27).

**Sex**		***n***
Male		8
Female		19
**Age**		
18–34		4
35–44		5
45–54		5
55–64		7
65–74		5
75+		1
**Self-reported ethnic or cultural group**^a^		
Arabic		1
Black		2
French Canadian or Quebecer		8
Visible minority		1
White		15
**Type of region lived in**		
Urban		21
Rural		6
**Education level completed**		
Technical or vocational school		4
College		5
University		18
**Employment status**		
Full-time work		2
Part-time work		3
Parental leave		1
Pension		6
Student		2
Temporary disability/workers’ compensation		5
Permanent disability		8
**Annual income vs. minimum required to meet basis needs**^b^		
Above		19
Below		8
**Self-reported diagnosis/types of chronic pain**^c^		
Musculoskeletal chronic pain		24
Fibromyalgia		6
Neuropathic pain		7
Complex regional pain syndrome		2
Migraine		2
Postconcussion syndrome		1
**Number of physiotherapy settings consulted**		
One	4	
Two		10
Three or more		13
**Types of physiotherapy settings consulted for chronic pain**		
Only private settings		13
Only public settings (including hospital, rehabilitation center, etc.)		2
Both private and public settings		12

^a^In response to the open-ended question: “From your perspective, which ethnic or cultural group do you identify with?”

^b^Minimal annual income for a person to be able to meet basic needs as established by the government of Quebec in 2020 was $20,767.

^c^Some participants self-reported more than one diagnosis/type of chronic pain.

### Data analysis

All interviews were first transcribed verbatim by a professional transcriber. The transcripts were then verified for exactness by the first author. Concurrent data collection and analysis were used, which allowed us to make minor adjustments to our interview guide to explore specific elements as well as to use theoretical sampling to recruit participants with various profiles and experiences.^45^ We conducted a reflexive thematic analysis^51^ with an inductive approach using the constant comparative method.^45,51,52^ This reflexive approach acknowledges the active role of researchers in knowledge creation, enabling critical examination of how our positioning influenced the analysis while maintaining analytical rigor.^51^ The collaborative and iterative nature of the approach, involving multiple team members in coding and theme development, helped ensure interpretations remained grounded in participants’ accounts while generating meaningful patterns of shared meaning that could enhance understanding of participants’ needs regarding physiotherapy services. The analysis was performed using NVivo v14.^53^ To start, the first author (J.G.H.) inductively coded the verified transcripts. The verbatim text of the first interview was also coded by the second author (A.F.) and the senior author (A.H.), who has a rich experience in qualitative research and thematic analysis. Codes were then compared and discussed between the three coders. Minor adjustments were made to the codes and to the coding process. Throughout the rest of the coding process, meetings were held with team members to discuss the coding and make adjustments as necessary. The two study patient partners were consulted on three occasions to discuss the codes obtained and the reflexive memos developed by the first two authors. Conceptual maps were also created by the first author and discussed with members of the team, to group together codes and ideas to identify themes. This iterative and collaborative process ensured that codes were grounded in participants’ words and experiences. These codes were then organized into broader categories based on shared or implicit meanings, from which themes and subthemes were developed to offer a more comprehensive understanding of the data.^54^ The quotes included to illustrate the themes and subthemes were translated from French to English by a bilingual individual and verified by a native English speaker. The translation was performed at the final stage of the writing process in order to preserve the original meaning of participants’ statements for as long as possible throughout the analysis.

### Research team

The research team was led by a PhD student with clinical experience as a physiotherapist working with people living with chronic pain in the private physiotherapy clinic he owns (J.G.H.). A kinesitherapist (physiotherapist in France) completing a master’s degree in clinical research was also part of the research team (A.F.), as well as three physiotherapists and researchers with expertise in ethics and chronic pain (A.H.), access and organizations of services for people living with pain (K.P.), and chronic pain education and management (T.H.W.). Another researcher with expertise in primary health care organization and program evaluation (I.G.) and two patient partners with various experiences using physiotherapy services completed the research team (C.C. and F.L.).

### Rigor and reflexivity

Trustworthiness was established through multiple strategies addressing four criteria: credibility, transferability, dependability, and confirmability, as recommended by Ahmed.^55^ To ensure dependability, all decisions concerning the course of the study were recorded in an audit trail. After each interview, an analytic memo was written by the interviewers to summarize the context of the interview and the main content addressed, to suggest modifications to the interview guide, and to write reflections on the interview. Throughout the study, interviewers kept a reflective diary to record their impressions and reflect on their interpretation of the collected data, enhancing confirmability. Reflexive discussions were held regularly during the analysis process, allowing team members to engage critically with the data and reflect on their own positions and experiences, further supporting credibility. The inclusion of two patient partners enriched all stages of the study, helping to anchor interpretations in the lived realities of individuals with chronic pain. Though these partners brought valuable insights, they were also aware of the subjectivity of their own experiences and that others may perceive pain and physiotherapy differently.

Most authors had expertise or experience with chronic pain, whether through research, clinical practice, or lived experience. They view chronic pain as a subjective and multifaceted phenomenon, shaped by biological, psychological, and social factors, and recognize its broad impact on individuals’ lives. This shared understanding of pain informed the analytic lens, emphasizing the subjective and contextual nature of participants’ experiences, which supports transferability through detailed contextual description.

The first author (J.G.H.), who led the recruitment, data collection, and analysis, had significant clinical experience with individuals living with chronic pain. Through this clinical experience, he had become aware of the importance patients place on therapeutic relationships, education, and longer sessions, as well as the frustration many experience regarding lack of effective treatment options or access to resources. He was mindful that these assumptions could influence how he conducted participant recruitment and interviews. He also acknowledged the potential impact of these assumptions on his interpretation of participants’ narratives. Maintaining a reflexive journal throughout the research process enabled him to critically examine these assumptions and their possible influence. Additionally, analytic memos and team discussions supported a reflexive approach, helping to distinguish personal perspectives from those of the participants. This ongoing reflexive process enhanced the team’s and first author’s awareness of their own underlying assumptions regarding chronic pain, thereby supporting a more balanced and critically informed interpretation of participants’ narratives. This process further contributed to a more robust and nuanced understanding of the needs and themes by encouraging the reconsideration of preliminary interpretations and the exploration of alternative meanings, thereby avoiding a narrow perspective shaped solely by our clinical and research experiences. In addition to this reflexive stance, the team’s overall knowledge of physiotherapy services and chronic pain, further strengthened by the involvement of two patient partners, helped situate participants’ narratives as well as the needs and themes identified within a physiotherapy context.

## Results

**Figure 1:**
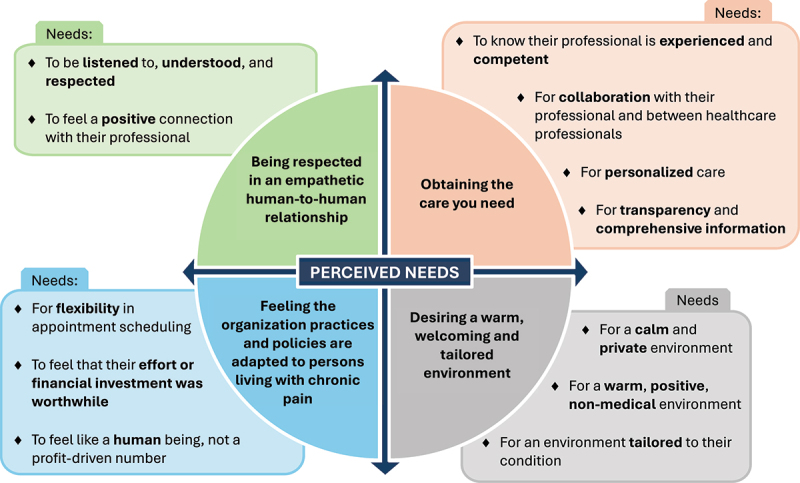
The four interconnected themes and perceived needs (sub-themes) related to physiotherapy service.

Four interconnected themes related to patients’ perceived needs regarding physiotherapy services were identified from our thematic analysis (see Figure 1): (1) being respected in an empathetic human-to-human relationship; (2) obtaining the care you need; (3) desiring a warm, welcoming, and tailored environment; and (4) feeling the organization practices and policies are adapted to persons living with chronic pain. Each of the themes encompasses various perceived needs categorized as subthemes.

### Theme 1: Being respected in an empathetic human-to-human relationship

This first theme reflects participants’ needs related to an empathetic interpersonal patient–professional relationship. Two subthemes are encompassed in this theme: the need to be listened to, understood, and respected and need to feel a positive connection with their professional.

#### Need to be listened to, understood, and respected

All participants in our study expressed the need to be listened to, understood, and respected through an empathetic human relationship. As mentioned by one of the participants, living with chronic pain can be an unpleasant and discouraging emotional experience. She mentioned the need to feel that the professional took time to listen to her:

I was very lucky […] so many times they saw me emotional and crying because I was in pain, crying because I was discouraged, and they put the exercises aside and said, “Let’s take the time to talk! Come sit down and we’ll talk.” It wasn’t just like … it was like important for me to feel comfortable there. It was spontaneous. (A08, woman, 35–44 age group, consulted in private settings)

Some participants also felt disrespected and judged by their physiotherapy professional, and they expressed the need to be understood and respected. One participant specifically reported feeling neither believed nor respected by her professional, a situation she attributed to the perceptions professionals have toward persons experiencing chronic pain.

In physiotherapy, it’s dictated, it’s in books. There are terms, there are positions, there are degrees, there’s … acceptability and then when they judge that what you’re saying isn’t credible, well, they mark it in the report and you are seen as a weirdo who doesn’t seem to want to help herself. (A28, woman, 55–64 age group, consulted in private settings)

Many participants indeed reported feeling misunderstood, not believed, and stigmatized by those around them, including the professionals. Conversely, because participants turned to their professionals with the hope of finding help, they stressed the need to really feel heard and understood by them.

#### Need to feel a positive connection with their professional

Some participants raised the need to feel a positive connection with their professional to cope with negative feelings often brought on by living with chronic pain. As one participant stated, laughing with the professional helped to build the relationship and feel more relaxed:

I found that with my therapist, he might tell me a joke and we’d have a laugh, I find that there is more of a connection and that makes the therapist listen to you. […] During 4 months at [name of a second physiotherapy setting], no one has ever made a joke, no one has ever laughed there. […] Everyone is serious and … it’s very rigid. […] If you’re in a rigid atmosphere again, it doesn’t help you try to relax there. (A25, woman, 18–34 age group, consulted in private and public settings)

Many participants also highlighted various human qualities they appreciated from their professional, such as devoted, honest, warm, attentive, generous, cheerful, humble, kind, professional, etc. They felt that these qualities enriched their relationship with their professional, making it more positive and warm. As one participant said regarding the professional she consulted:

She’s very professional. She really wants to help. And she’s always in a good mood [laughs]. […] She has a great joie de vivre, she’s always very welcoming. […] And as she’s always cheerful—it’s contagious. It helps boost morale if it’s low. I think it’s perfect. […] She’s a calm person. […] I know she’s always cheerful, always smiling and in a good mood, and happy to see us. It brings calm, it stimulates […] that the person is warm, you know, it’s essential. (A22, woman, 55–64 age group, consulted in private settings)

Thus, for many participants, the qualities mentioned above, as well as the feeling of having a positive connection with the professional, contributed to the empathetic and human relationship they needed to cope with the negativity they often felt by living with chronic pain.

### Theme 2: Obtaining the care you need

This second theme encompasses the participants’ perceived needs related to the care they seek. It regroups four subthemes: need to know their professional is experienced and competent, need for collaboration with their professional and between their health care professionals, need for personalized care, and need for transparency and comprehensive information.

#### Need to know their professional is experienced and competent

Many participants raised the need to consult a physiotherapy professional they felt was competent, especially for working with people living with chronic pain. As one participant mentioned:

So I really looked around for someone who had a lot of experience, […] someone who knew what fibromyalgia was and who wasn’t going to give me exercises that were going to hurt me. (A14, woman, 35–44 age group, consulted in private and public settings)

Most participants felt their professionals were competent if they had several years of work experience, though others valued more the condition-specific work experience, professional certifications, age, session rates, and type of setting. For example, one participant felt she could put more trust in the competencies of a professional working in a public setting compared to a private setting.

Because when they’re hired by the hospital, you know that they’re really people with the right qualifications. And that makes me feel a lot more secure. In the private sector, I don’t know. They may have a diploma on the wall, but you don’t really know … if you can trust them 100%. […] I think you have to trust the staff on site, and our needs aren’t all the same, are they? Okay, we need reassurance, we need someone competent. But I think that in the public, everyone is competent, have their skills, that’s for sure. (A20, woman 65–74 age group, consulted in public settings)

Most participants mentioned looking for an expert professional who could and would understand the complexity of their chronic condition. Many had consulted several professionals, driven by the hope of feeling better. It was therefore important for them to find the right professional, based on the criteria they considered important. They also wished information was available to guide them, as this participant mentioned:

I can’t tell you how important it is to have the right professional for the right problem. It’s so vital. […] There are so many big differences […] in the experience of physios, but it’s incredible. Why isn’t there more information? […] It’s vital information that we should know. (A21, woman, 45–54 age group, consulted in private settings)

Overall, for most participants, competence went beyond just technical skills. Participants acknowledged the complexity of their condition, often emphasizing the uniqueness of their body and pain, and sought a professional who could genuinely understand them and their experience of pain.

#### Need for collaboration with their professional and between their health care professionals

Many participants wanted to collaborate with their professional to set goals, elaborate the treatment plan, and ensure they received interventions that account for their pain, their abilities, and their overall realities:

When I saw, for example, at [rehabilitation center #1] afterwards, there was an intervention plan where I could participate and name what was important for me. I really appreciated that. I felt like I was participating in my rehabilitation, whereas there [talking about another physiotherapy setting], I felt like I was less involved. […] It was more like I was enduring my physiotherapy instead of participating in it. (A27, woman, 18–34 age group, consulted in public setting)

Participants also stressed the importance of having health care professionals collaborate and communicate with each other to benefit from a more comprehensive and holistic approach. They wanted better collaboration between health care professionals to receive consistent information and avoid repeating the same information over and over again.

I think teamwork is really lacking. […] All the physios work in silos. They could all talk to each other, and I think that would help everyone […] work more as a team so that the patient is more at the center and doesn’t feel like he’s being thrown from one side to the other. […] Each professional had their message, but all the messages contradicted each other. So you listen to who? […] You want to stop being in pain, so you believe what the person tells you, but then the professionals contradict each other, so you’re lost! (A25, woman, 18–25 age group, consulted in private and public settings)

As one participant also noted, it should not be the patient’s responsibility to bridge the gap between health care professionals:

You’re not the doctor. If you’ve been diagnosed with XYZ, it’s not up to you to explain to the physio what’s wrong with you. (A21, woman, 45–54 age group, consulted in private settings)

Therefore, participants mentioned they need a better collaboration between all stakeholders to feel more involved in their care, to receive comprehensive and consistent information, and to avoid the burden of repeating everything to each new professional they encounter. These statements may also highlight the power imbalances often present in patient–professional relationships and an underlying desire to move toward a more egalitarian relationship where their voices are heard and valued.

#### Need for personalized care

Many participants emphasized the need for personalized care. Participants frequently highlighted the complexity of their pain and condition, emphasizing their individuality and the need for care tailored to them. As one participant stated:

Because everyone is different. The approach should be different for each individual and not mechanical, not technical like in books, because there’s beyond that. (A28, woman, 55–64 age group, consulted in private settings)

Some participants felt their physiotherapy professional treated their pain without trying to really understand them as a whole person, which led to receiving services that were not personalized:

She didn’t really listen to my body. She went there a little bit, she did standard exercises, so, “First you have a shoulder problem, you have to do this and this and this exercise.” […] The shoulder problem is the same for all patients. So, there wasn’t really any … listening or … adaptation to my body, which is quite particular. (A14, woman, 35–44 age group, consulted in private and public settings)

To feel they received personalized care, many participants reported the need to receive different types of interventions: manual therapy, exercise, education, and group sessions. However, participants’ opinions on specific interventions varied. For example, many participants emphasized the importance of manual therapy, whereas for others, being touched was perceived as an aggression. Some participants felt that a balance between exercises and manual therapy was important.

As for exercises, most participants mentioned the need for a simple, well-taught, and clear exercise program, supported by a reminder checklist:

She made me practice. She explained things and always gave me a sheet to remind me how often, how many times, and it was very clear, well structured, very good. I felt supported in that. (A20, woman 65–74 age group, consulted in public settings)

Although most participants wanted direct one-to-one monitoring during the whole time they did their exercises, others found it appropriate that their professional left them alone to go see other patients, as long as instructions were clear and supervision still adequate. However, as one participant noted, being left alone during exercises may lead to uncertainty about how to do the exercises:

We were two or three at the same time with the same physiotherapist but separated by little curtains, […] it wasn’t one-on-one. So it was more just having her explain the exercises to me one by one, waiting for her to come back to do the other exercise. […] I got the impression that I was maybe doing them a little bit out of order, but I don’t know, I didn’t have any feedback, she wasn’t there. (A07, woman 35–44 age group, consulted in private and public settings)

In addition, for many participants, education was perceived as important to better understand their condition and to know how they could help themselves to get better.

To educate myself, […] it really changed my life. … You know, at first it wasn’t a big deal, but then I integrated that knowledge and it really changed my life. (A23, woman 18–34 age group, consulted in private and public settings)

Many participants also raised the need to have access to group sessions, mainly by video conference. They saw it as a way to access physiotherapy at lower cost, to exercise and be educated, as well as to participate in an exchange about their reality and share resources with others.

Overall, participants had specific and various needs related to the possible interventions offered during a physiotherapy session. These needs sometimes greatly differed from one person to another; hence the need for personalized care.

#### Need for transparency and comprehensive information

Some participants also highlighted the need to have comprehensive information about their care. They wanted to see the full picture. This helped them better understand what had been accomplished and what was going to happen next. Some participants mentioned the need to know what was really going on and whether their condition could truly improve.

I don’t know if sometimes they want to make a bit of money out of us and they tell us that the case can be cured. […] If they told us, “Well, there’s nothing else to do or your thing is broken,” or I don’t know what, but I find that … they don’t tell us all the time. […] I think they should tell us, “This is irrecoverable” so we’d get an idea, or tell us, “We can reduce it to 50% at best.” I like to know where I’m going. (A24, woman, >75 age group, consulted in private settings)

This need for transparency was also reflected by their desire to have a better idea of the number of sessions to expect. Some of them had the impression that the professional was continuing the sessions when there was no further progress.

That’s a turnoff for me. I don’t want a lifetime program. So I want a timetable. I’d like to have a timetable, how long he thinks. Of course it’s not an exact science, but how long he thinks it will take to solve this problem. (A16, woman 65–74 age group, consulted in private and public settings)

For others, discharge from physiotherapy treatment came suddenly. They sometimes felt left to themselves, despite the pain or functional limitations that were still present. Therefore, many participants wished they could have access to a follow-up appointment from time to time. Otherwise, as one participant mentioned, once they have been discharged from physiotherapy, people go off on their own to try and make further progress, but they often have to return to physiotherapy.

You tell yourself, “It’s chronic so get used to it” […] until sometimes you get tired of it and you start again. “Well, go back to physio to see if we can improve.” It’s a wheel that never ends! (A24, woman, >75 age group, consulted in private settings)

Participants felt the need for transparency and comprehensive information that would allow them to better anticipate the upcoming steps.

### Theme 3: Desiring a warm, welcoming, and tailored environment

The third theme identified concerns participants’ needs related to consulting physiotherapy services in a warm and welcoming physical environment. It contains three subthemes: need for a calm and private environment; need for a warm, positive, and nonmedical environment; and need for an environment tailored to their condition.

#### Need for a calm and private environment

Several participants raised the need for privacy and confidentiality by seeing their professional in a closed, private room instead of an open room; that is, a space shared by several people at a time. As this participant noted, living with chronic pain can evoke a range of emotions that one may prefer not to share with those around them:

When you see a physio, the memories I’ve had, many times I’ve cried my eyes out, and with the pain of seeing everyone there watching you. I’m not … at least a minimum of privacy to be able to manage your suffering on your own, that no one is obliged to see you suffer, personally, I find that important. (A06, man, 55–64 age group, consulted in private settings)

Some participants who had received physiotherapy services in an open room also found it harder to concentrate and were less comfortable communicating with their professional. Nonetheless, participants’ preferences for the type of room also depended on the context and the timing of the rehabilitation, as this participant explained:

When I arrived at the center, yes, there were curtains, they weren’t closed rooms, but the context was different. I was on another level and I felt more comfortable talking about certain things or asking questions to the person I was with. […] It was like I was on another level in my life, another level in my situation, my state of health. (A19, woman, 45–54 age group, consulted in private and public settings)

For some participants, having a quiet, private place allowed them to better communicate with their professional and to be more comfortable. However, this need varied among participants, and at times some of them wished they had an open room to socialize with other patients.

#### Need for a warm, positive, nonmedical environment

Beyond the type of room used (open or closed), some participants mentioned the need to receive physiotherapy services in an environment characterized by a warm, contemporary decor, moving away from a medical look. As one participant explained, a “medical-looking” environment can affect one’s mood:

You know, you have to relearn how to live, you have to relearn how to socialize because of pain. You have to learn to laugh again, […] and you’re in a clinic, the walls are beige and all you see on the wall are pictures of injuries. It’s not very encouraging. (A28, woman, 55–64 age group, consulted in private setting)

Some participants perceived an environment filled with light, color, plants, and beautiful photos or paintings as more positive and welcoming:

Less … that’s it, less medical, […] it brings calm, it brings … I think it stimulates. That’s it, it stimulates the sight positively. […] Aesthetics, I think it’s positive. […] Getting out of the medical, it’s like … it’s warm. (A22, woman, 55–64 age group, consulted in private settings)

Some participants reported that a medical environment was often associated with long wait times and disappointments related to their chronic pain. They therefore felt more welcome and encouraged when in a nonmedical environment.

#### Need for an environment tailored to their condition

Some participants also perceived the need to receive physiotherapy services in an environment adapted to their condition. For example, this participant mentioned needing a spacious environment to be able to move when in pain.

It was a big room. […] If I was in pain, I could afford to walk around the room because it was a big, open room, whereas the other office was a small office upstairs. (A19, woman, 45–54 age group, consulted in private and public settings)

Some participants considered it unacceptable to obtain care in an environment that was overloaded, ill-suited, or not dedicated to physiotherapy:

Because there’s a lot of stuff in the office that isn’t related to physiotherapy. A lot of stuff that has nothing to do with it. […] They’re not equipped with permanent facilities. They should be, because the pain clinic is a clinic that is there permanently. […] It’s better when you go in an environment that’s clean and really dedicated to physio. […] It’s as if they’re taking you as a last resort. […] We’re stuck with you here, we’re going to find you a little corner [laughs] to try to give you an exercise. (A16, woman, 65–74 age group, consulted in private and public settings)

Therefore, in addition to the look and design of the environment, participants considered the environment layout important to feel comfortable during sessions and as if they were being taken seriously.

### Theme 4: Feeling the organization practices and policies are adapted to persons living with chronic pain

This fourth theme relates to participants’ needs to feel that organizational practices and policies are adapted to people living with chronic pain. It includes three perceived needs: need for flexibility in appointment scheduling, need to feel that their effort or financial investment was worthwhile, and need to feel like a human being, not a profit-driven number.

#### Need for flexibility in appointment scheduling

Many participants emphasized the need to choose their day and time of appointment to better align with their daily activities, work, other appointments, and overall well-being. For some participants, online appointment booking was a way of making bookings easier and more flexible. As mentioned by one participant:

So it’s easy, in terms of management, to find the best time depending on other medical appointments, physical activities, […] I know that when I do physio, not always but it often happens that the next day I’m more tiredm […] so I can put it wherever I want, depending on what I have, […] It’s really effective. There are less intermediaries. (A22, woman, 55–64 age group, consulted in private settings)

However, this flexibility in appointment scheduling was not possible for all participants. This next patient reported experiencing imposed appointment times:

It was practically set in stone: “I can see you there! But is it possible the next day? No, no it’s not possible, it’s there!” meaning it’s there or nothing, […] there was no flexibility, it was rigid. (A15, man, 65–74 age group, seen in private settings)

The words of other participants, who had consulted in private clinics, also reflected a lack of flexibility such that they felt they were being unfairly charged for situations beyond their control. For example, one participant had to pay for cancellation fees when it was the pain that had prevented him from going to an appointment:

Because you couldn’t have imagined that the pain would be greater that day than the day before. They really wanted you to give at least 24 hours’ notice. Still, I thought it was a lack of understanding of what we were going through with chronic pain and our limitations and everything. (A10, man, 35–44 age group, consulted in private and public settings)

In addition, some participants found it difficult to book appointments with more experienced professionals. For example, this participant explained the difficulty of seeing a specific professional at a private clinic, while working to afford the services.

Physiotherapists who are specialized don’t work evenings. What kind of nonsense is that? […] Why would I cut my working hours? I need the money to pay for it. (A17, man, 35–44 age group, consulted in private settings)

According to several participants, more flexibility in scheduling appointments would better suit their reality, because they often had to juggle between work and medical appointments, in addition to dealing with the unpredictability surrounding chronic pain.

#### Need to feel that their effort or financial investment was worthwhile

Several participants found that the duration of their physiotherapy sessions was too short considering the complexity of chronic pain. As one participant stated regarding the professional role:

In half an hour, you have to get to know the file, do your treatment, finish your file, and then move on to another client. Well, maybe that’s normal when you come in for a sprained pinky finger. But for something that causes chronic pain, where the patient may need more guidance, […]half an hour may not be enough. (A17, man, 35–44 age group, consulted in private settings)

For some, like this other participant, going to physiotherapy ultimately required a lot of effort for little treatment time:

Traveling to get there, you get there, there you talk for 5, 10 minutes. “Lie down on the table.” You’re treated for 10 minutes and then you leave! It’s a lot of effort for 10 minutes. […] I was like, “I’m wasting my time, I’m tired, I don’t feel like being there.” And on top of that I feel like I’m wasting my time, so it was doubly frustrating! […] While you’re there, you’d do an hour, but it’s too expensive. (A25, woman, 18–34 age group, consulted in private and public settings)

Furthermore, considering that most participants consulted for physiotherapy in the private sector, the cost of sessions was a major barrier limiting the number or duration of sessions. Many participants would have liked to attend physiotherapy more often, for longer sessions, or over a longer period of time, but they often had to wait before returning due to financial reasons.

In the private sector, it’s all about costs. […] The service is great, but the costs mean that it can become difficult to respect the right treatment frequency for you to evolve. (A25, woman, 18–34 age group, consulted in private and public settings)

Unfortunately, some participants felt that they had spent a lot of money without seeing the expected results in terms of outcomes. This made them feel like they did not get the expected value of treatment for their money. This next participant explained that she could probably have done the same at home:

Paying $85 to do exercises for 30 minutes, when in theory I could do them at home, bothers me a bit. (A01, woman, 18–34 age group, consulted in private settings)

Given the complexity of chronic pain as highlighted by participants and the financial burden of private-sector consultations, some participants felt it was important to ensure the sessions were worth the investment, seeking longer appointments and a sense that neither their time nor money was being wasted.

#### Need to feel like a human being, not a profit-driven number

Some participants mentioned they felt they were seen as a number and not as a human being:

You can well say that the public [settings] are too rigid, you’re a number, you really don’t feel like a human being who is in pain right now, you’re just like another person on the waiting list, and then they just want to finish with you to get another person in. (A25, woman, 18–34 age group, consulted in private and public settings)

Many participants who went to the private sector felt like the primary intention was to see lots of patients to make money:

It’s, like, it’s really just a french fries factory. They were there just to make money, and there wasn’t much contact with the client. (A14, woman, 35–44 age group, consulted in private and public settings)

For the participants, this sense of being human was closely connected to the quality of the attention given by the physiotherapist and the organization. As one participant said:

Presence, an authentic presence. You know, we are all human beings. (A28, woman, 55–64 age group, consulted in private settings)

## Discussion

Our study has drawn on the experiences of some people living with chronic pain to better understand their needs. Our thematic analysis resulted in the identification of four overarching themes related to the perceived needs of people living with chronic pain regarding physiotherapy services.

The broad spectrum of perceived needs identified in our study echoed several needs previously described in the literature^31^ underscoring the complex and multifaceted nature of needs related to physiotherapy. Our findings suggest that these needs are deeply interconnected and shaped not only by individual experiences but also by broader organizational and systemic forces within health care.

### Interconnectedness

Perceived needs are inherently subjective and may vary from one individual to another. However, recognizing chronic pain as a complex experience that can significantly impact physical, psychological, and social well-being helps to explain why people living with chronic pain may share similar perceived needs. Confronted with a real but often invisible and nonobjectifiable pain, people living with chronic pain often encounter skepticism from those around them.^56,57^ Combined with intense pain and functional limitations that affect their daily lives, work, and social interactions,^9,58^ many individuals may feel stigmatized, misunderstood, socially excluded, and rejected.^9,36^ It is therefore understandable that these people might express a strong need for an egalitarian therapeutic relationship marked by empathy and humanity, to help them feel believed, improve their self-confidence, and maintain their “social self,” which can be profoundly threatened by the experience of living with chronic pain.^58,59^ Interestingly, findings of recent qualitative studies emphasizing the importance of empathy and positive communication^60–64^ also encompassed elements related to another theme identified in our study—obtaining the care you need—reinforcing the idea that both the therapeutic relationship and the professional’s expertise and technical skills are important in delivering quality care.^65,66^ Indeed, participants in our study frequently expressed their need for a competent therapist with expertise in chronic pain, while emphasizing that such expertise must be accompanied by strong interpersonal qualities to foster effective listening and communication. As Mengshoel et al. noted, patients with chronic pain found their experience more meaningful when they are listened to and taken seriously, as well as when they received a tailored exercise program, not a one-size-fits-all program.^64^

Our study also identified needs related to the physical environment of physiotherapy settings, an aspect that few studies have addressed to date.^31^ Only one qualitative study exploring the physiotherapy experience of patients with chronic low back pain reported that some patients appreciated the support and camaraderie offered by open rooms shared with other patients.^63^ In a metasynthesis on satisfaction in outpatient musculoskeletal physiotherapy (not limited to chronic pain), Rossettini et al. reported that the physical environment was important for patients’ comfort (e.g., being in a private room) and also for patients’ safety,^67^ an element that was not found in our study. Many participants in our study explained the negative feelings they often felt in a medical-looking environment and how busy and loud environments affected their concentration and their communication with their professional, emphasizing their need for a calm and nonmedical environment. Similar results were found in other studies on health care environments, where environments with single-bed, peaceful rooms decorated with colored walls and artwork were linked to improved patient–professional communication^68^ and interaction,^69^ as well as better outcomes.^70^ Furthermore, though this was not explored in our study, the physical environment can impact health care professionals’ well-being, thus affecting their interactions with patients.^71^ These findings reflect the interconnection between the physical environment and the therapeutic relationship, suggesting that despite goodwill and strong communication skills from professionals, a good therapeutic relationship may be difficult to develop if the physical environment is not conducive to it. These interconnected needs, though often expressed at the interpersonal level, are also profoundly shaped by the broader organizational and systemic context in which physiotherapy services are delivered.

### Driven by organizational and systemic forces

The fourth theme we identified encompassed needs that were closely linked to the organization of care. This theme not only reinforces the interconnectedness between the needs described above but also illustrates how the needs perceived by individuals living with chronic pain in relation to physiotherapy services are shaped by organizational and systemic forces embedded within our health care system. Though several studies have explored the needs and expectations of individuals living with chronic pain regarding physiotherapy services, few have specifically examined how these needs are influenced by broader organizational and systemic logics.^31^

One salient perceived need identified in our study is the need to feel like a human being, not a profit-driven number. Some participants, particularly those who had attended a private clinic, linked aspects of their physiotherapy experience, such as short session times, high costs, and lack of personalized treatment, to the lucrative intent of the clinic. Some even compared the experience to being treated in a factory, where productivity takes priority. This feeling was also noted in an Australian qualitative study on patients’ experiences in physiotherapy, where participants described feeling like they were in a “sausage factory” or on a “supermarket shelf.”^72^ Though this factory-like treatment was mentioned when discussing private clinics, in part linked to their fee-based services, it could also apply to public settings, where professionals are often pressured to meet output targets.^73^

This perception of being treated like a number in a factory echoes long-standing critiques of a biomedical tendency to view the body as a machine to repair.^74–76^ Though increasingly recognized as outdated, the biomedical model remains deeply embedded in physiotherapy practice, within physiotherapists’ thinking, words, and working environment.^74,76^ Unfortunately, such a model reinforces power asymmetries in therapeutic relationships^77^ and impacts physiotherapists’ perceptions of chronic pain,^78^ which could contribute to the stigmatization of people with chronic pain^10^ and limit the capacity to adequately respond to their needs. Despite growing evidence supporting the use of the biopsychosocial model, numerous studies continue to document barriers to its implementation in physiotherapy practices.^79–81^ Yet, a biopsychosocial model emphasizes active listening and understanding of the person as a whole, two elements closely aligned with the need to be heard and understood expressed by most participants in our study.

Over the past decades, Western governments have increasingly embraced neoliberal policies grounded in individualism and privatization, profoundly influencing the organization of health services, including physiotherapy.^76,82,83^ Neoliberalism is an ideology that frames health care as an individual private good rather than a public right, and in practice it manifests through policies that prioritize market mechanisms, deregulation, competition, and privatization, diminishing public funding and emphasizing individual responsibility over collective well-being.^82,83^ In our study, most participants had consulted in private clinics, not by choice but due to limited access to publicly funded services. This reflects a neoliberal market-oriented approach that tends to favor privatization. Though such models can raise equity concerns, these are primarily linked to insufficient public reimbursement and control, which shifts access from need-based to means-based.^82^ The lucrative nature of many private clinics also often translates into high fees and shorter appointments, as previously mentioned, and linked to some of the perceived needs identified in our study. Furthermore, the emphasis on individual responsibility rooted in neoliberal principles resonates with participants’ reported need for collaboration and support. By framing health as a personal matter, neoliberal governance tends to downplay collective responsibility and promote independence.^83^ This individualistic lens also assumes that policies rooted in a neoliberal mindset will benefit everyone equally.^83^ This perspective reflects how neoliberal policies often lack attentiveness and responsiveness to people’s actual needs and may, in fact, generate new ones. As raised by Praestegaard et al., neoliberalism and the medical gaze surrounding the profession influence physiotherapy services by shaping both the clinical environment and the ways in which physiotherapists act and communicate, often unconsciously producing “intelligible” patients who fit within the neoliberal logic of the organization and the broader system.^76^ This dynamic, also present in the public sector,^84,85^ undoubtedly shapes people’s needs.

Altogether, our findings underscore that perceived needs cannot be understood, or addressed, in isolation from the organizational and systemic structures that shape them.

### Moving forward: Multilevel action needed

Adequately addressing the needs of people living with chronic pain may therefore require action at the organizational and health care system levels. If physiotherapy services and health care systems do not consider and adapt to the specific needs of people with chronic pain, it may be difficult, if not impossible, to meet those needs effectively. This calls for a critical reflection on current physiotherapy practices to challenge taken-for-granted assumptions, often shaped by neoliberal influence, that may disadvantage individuals with chronic pain. For instance, a recent study found that websites of many physiotherapy clinics primarily featured visual and textual content aimed at individuals with sports injuries, whereas very little, if any, content was directed toward those living with chronic pain.^44^ Attracting individuals with “simple” or easily managed conditions enables clinics to maintain the appearance of efficiency and positive patient outcomes, thereby supporting patient satisfaction, repeat visits, and referrals, all factors that ultimately contribute to clinics’ profitability.^44,76^ This example highlights significant issues of equity and representation that individuals living with chronic pain often face, stemming from deeply rooted practices that may seem harmless but are, in fact, problematic. Conventional models of care, typically oriented toward treating an injury or an objectively measurable condition, might therefore be likely ill-suited to the complexity and subjectivity of chronic pain.^21,86^ As suggested by Christopoulos and Peter, invisibility, a key characteristic of chronic pain, forms a cornerstone of the lived experience and suffering of individuals who live with it.^87^ This invisibility undermines care quality and the therapeutic relationship, particularly within a health care system focused on addressing objectively identifiable problems.^87^ It significantly shapes patients’ suffering and, in turn, reinforces the invisible nature of their pain.^87^ Thus, a model of care that prioritizes patients’ narratives and lived pain experiences, such as narrative-based medicine^87^ or the multimodal assessment of pain model,^86^ may foster a more comprehensive and compassionate approach to health care for individuals living with chronic, and therefore invisible, pain.

Finally, some individuals, particularly those in vulnerable situations, may struggle to identify, express, and have their needs heard or well understood,^33^ resulting in their needs being contested by experts and authorities.^88^ For meaningful changes to occur, it is therefore crucial that decision makers and policymakers recognize and interpret perceived needs at their true value; that is, as genuine needs that they have a responsibility to try to meet and not “just” as someone’s desires or preferences. To achieve this, continuing the efforts of recent years to involve health care users in decision making across education, research, and the health care system would be highly beneficial.^89^ Such involvement would ensure that people’s experiences are heard, provide a deeper understanding of their realities, and better respond to their needs. It would also enhance health care services by making them more compassionate and humane. Managers, decision makers, and policymakers must also recognize and understand the interconnectedness and influence of the macrolevel forces previously described, in order to better grasp their roles and responsibilities in responding to perceived needs. Such awareness is essential to implement organizational practices and policies that more effectively address these needs and to avoid falling into the trap of placing the responsibility for meeting patients’ perceived needs solely on therapists. Fundamentally, not only health practices and policies but also people’s needs and their experiences of pain are profoundly political.^33,90,91^

### Study limitations

Although we made specific efforts to diversify our sample, people participating in our study were predominantly women, White, highly educated, and from a higher income background. Most participants lived in urban areas and had consulted in private clinics for musculoskeletal pain. Our sample lacked sociodemographic diversity compared to broader populations of individuals living with chronic pain.^91^ This may stem from our inclusion criterion of prior physiotherapy consultation, because research shows that White women with higher levels of education and income and living in urban areas are more likely to use physiotherapy services.^92,93^ Barriers to accessing public physiotherapy services for people with chronic pain may have further limited diversity in our sample.^94–97^ As a result, our findings may not fully reflect the needs of individuals from underrepresented groups, including those facing socioeconomic or geographic barriers to care. Moreover, we chose to include only individuals who had consulted in physiotherapy, because our objective was to explore needs specifically related to a physiotherapy consultation. Consequently, the needs of those who face barriers, who were unable to access such services, were not captured in this study. Future research should explore the needs of these individuals, because they may differ significantly and highlight important gaps in responding to the needs of persons experiencing chronic pain. It is also important to note that our study included only individuals who accessed physiotherapy services in Quebec. Therefore, patients’ needs regarding physiotherapy services may differ in other contexts.

## Conclusion

In conclusion, our findings provide a deep understanding of the perceived needs of people living with chronic pain in regard to physiotherapy services in Québec. Our study highlights the multiple and interconnected nature of these needs and how they are profoundly shaped by organizational and systemic contexts. Our findings underscore the importance of moving beyond individual-level strategies to better respond to people’s needs, as well as to address macrolevel determinants, such as organizational policies and systemic forces that influence the responsiveness of physiotherapy services. Strengthening this responsiveness therefore requires collective efforts from clinicians, managers, and policymakers to create conditions that are not only effective but also equitable, humane, and attentive to the experiences of people living with chronic pain. Unless these underlying organizational and systemic logics are clearly acknowledged and addressed, attempts to meet patients’ needs through fragmented or isolated interventions are likely to remain unsuccessful. The inherently interconnected nature of these needs calls for a comprehensive, systemwide approach that recognizes how responding to one need inevitably depends on addressing others.

## Supplementary Material

Supplementary file_Interview guide_EN_Nov17_2025.docx
