# Supplementary material for: “You know, we’re all human beings”: A qualitative study on the perceived needs of people experiencing chronic pain with regard to physiotherapy services
Source: Can J Pain. 2026 Mar 4;10(1):2615474. doi: 10.1080/24740527.2026.2615474 (PMC12962706; doi:10.1080/24740527.2026.2615474)
Supplement: Supplementary file_Interview guide_EN_Nov17_2025.docx [file UCJP_A_2615474_SM0315.docx]

**Question 1: To begin, could you please tell me a bit about yourself and what led you to participate in this study?**

**Question 2: Could you tell me about your pain?**

*Prompts for the interviewer*:
- Onset of the pain?
- How has it evolved?
- Impact on their daily activities?
- Impact on their quality of life?
- Their perception and the perception of others regarding their pain?

**Question 3: Could you please tell me about the care you received and the healthcare professionals you have consulted in the past for your pain?**

*Prompts for the interviewer:*- Professionals / care settings consulted? Physiotherapy?
- Overall perceptions of the consultations or treatments received?
- Did they meet their needs? How do they define a “need”?
- Expectations regarding the consultations or treatments received? Progress of their condition in relation to the treatments received?
- Challenges or difficulties encountered within this care?
- Effects of these interventions?

**Question 4: For the next questions, I would like you to focus on physiotherapy. How would you describe your experience(s) with physiotherapy services?**

*Prompts for the interviewer:*- Reason for consulting? Type of setting consulted? Why this setting or this therapist?
- Type of treatment received? In-person? Telerehabilitation?
- What were your expectations when you consulted? And your needs?
- Degree of satisfaction with the experience?
- Positive / negative aspects / areas for improvement from the experience?
- How did the overall care experience unfold? Scheduling, intake, treatment rooms, available equipment, payment of sessions (if in private sector), post-treatment follow-up?
- Opinion regarding accessibility? Opening hours? Waiting time?
- Opinion regarding the quality of care received? Interpersonal relationships?
- Did the physiotherapist understand your pain?
- Did the care meet your needs or expectations?

**Question 5: Now, could you please describe your needs and expectations regarding physiotherapy services?**

*Prompts for the interviewer:*- Needs / expectations related to access? To the clinic environment? To how the clinic operates? To the therapeutic relationship? To the quality of care received?
- Influence of the type of physiotherapy professional (physiotherapist or physical rehabilitation therapist)?
- Ideal (dream) physiotherapy experience?
- What was missing in their care or what could have been improved?
- Elements that disappointed them or that they disliked? Or that they appreciated?
- Are there types of therapy or care they expected? Or that should be offered?

**Question 6: Thinking back on everything we discussed, what are your suggestions to improve physiotherapy care for people living with chronic pain?**
